# Supplementary material for: Complete microtubule–kinetochore occupancy favours the segregation of merotelic attachments
Source: Nat Commun. 2018 May 23;9:2042. doi: 10.1038/s41467-018-04427-x (PMC5966435; doi:10.1038/s41467-018-04427-x)
Supplement: Supplementary file 1 — Supplementary Information [file 41467_2018_4427_MOESM1_ESM.pdf]

Supplementary Information

Complete microtubule-kinetochore occupancy favours the segregation of merotelic attachments

Dudka et al.

Supplementary Figure 1

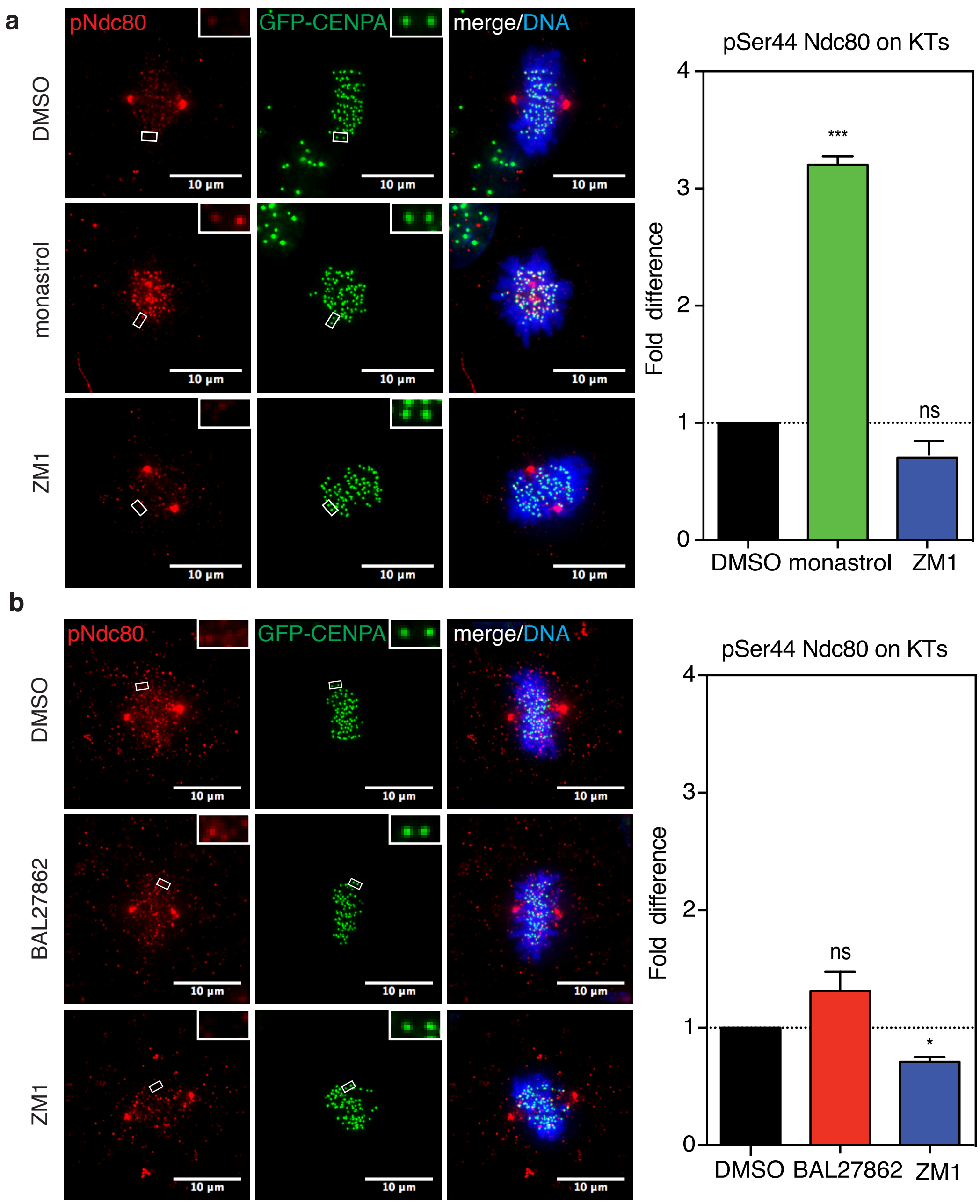

Supplementary Figure 1. Low inter-KT distances barely increase Ndc80 phosphorylation at KTs (**a**) Left panel shows immunofluorescence images of hTert-RPE1 GFP-CENPA cells treated with DMSO, 100 µ M monastrol or 2 µM ZM1 and stained with pSer44-Ndc80 antibodies (red); insets show exemplary sister-KT pairs; scale bars = 10 µm; right panel shows quantification of normalized ratio pSer44-Ndc80/GFP-CENPA after background subtraction, DMSO vs. monastrol $p < 0.001$ in ratio paired two-tailed t-test, error bars are s.e.m.. N = 3, n = 300-400 kinetochores. (**b**) Left panel shows immunofluorescence images of metaphase hTert-RPE1 GFP-CENPA cells treated with DMSO, 12 nM of BAL27862 or 2 µM ZM1 and stained with pSer44-Ndc80 antibodies (red); insets show exemplary sister-KT pairs; scale bars = 10 µm. Right panel shows quantification of normalized ratio pSer44-Ndc80/GFP-CENPA after background subtraction. Error bars are s.e.m.. N = 3, n = 280-600 kinetochores, ratio paired two-tailed t-test, ns = non significant.* represents $p<0.05$.

Supplementary Figure 2

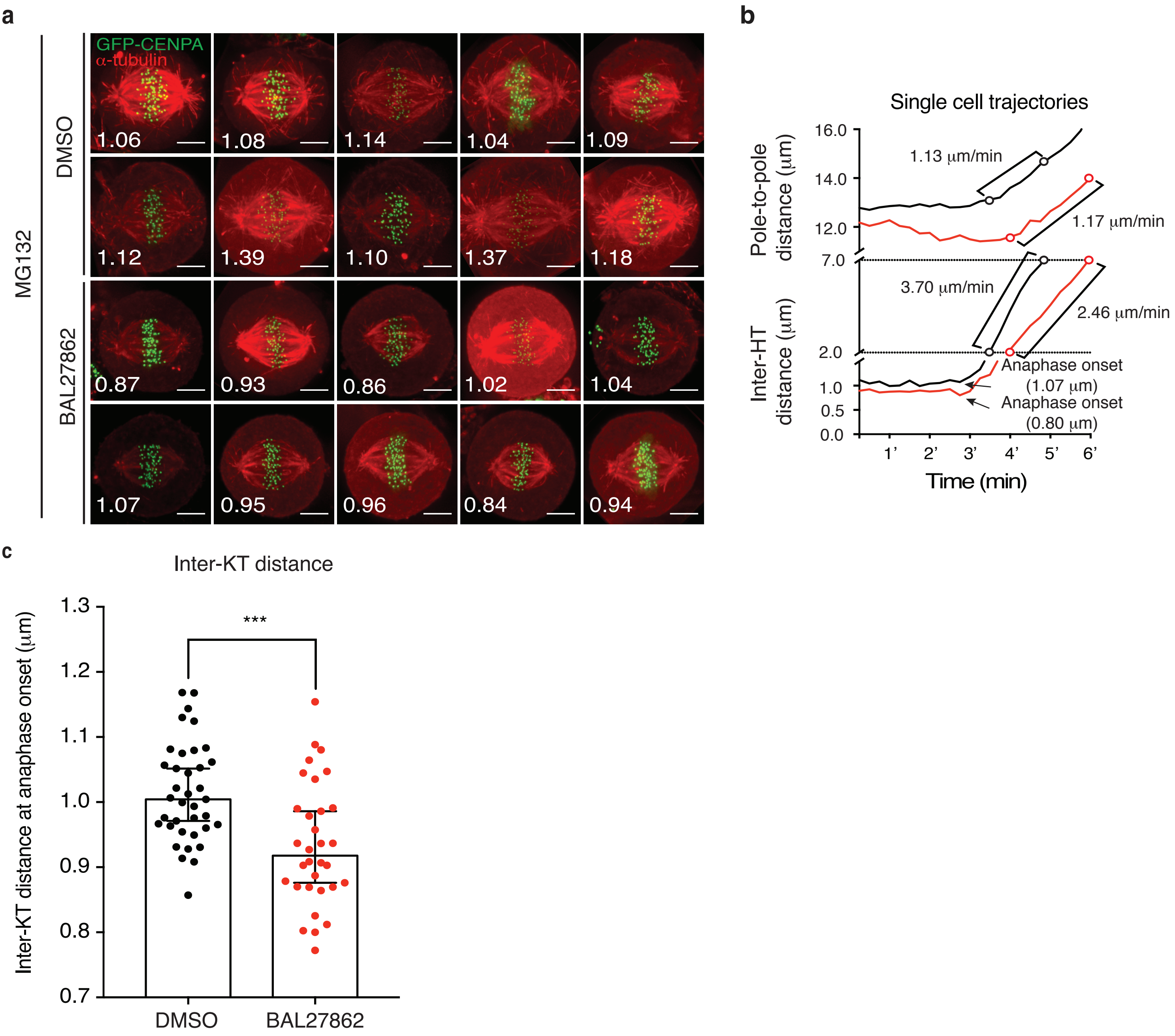

Supplementary Figure 2. Visualization of the effects of BAL27862 on astral MTs and anaphase spindle elongation (**a**) Immunofluorescence images of ten randomly selected hTert-RPE1 GFP-CENPA cells from a single experiment. Cells were treated in parallel with DMSO or 12 nM BAL27862, 10 µM MG132, and stained with α-tubulin antibodies (red). Scale bars = 5 µm. An average inter-KT distance per each cell is indicated in the left bottom corner. Note smaller asters in BAL27862-treated cells. (**b**) Single-cell trajectories of hTert-RPE1 Centrin1-GFP/GFP-CENPA cells treated with DMSO (black) or 12 nM of BAL27862 (red); average inter-KT distance before anaphase onset (bottom), the anaphase sister-KT velocities (centre) and spindle elongation velocities (top). T = 0 is arbitrary. (**c**) Quantification of inter-KT distances at anaphase onset of hTert-RPE1 Centrin1-GFP/GFP-CENPA cells; N = 4, n = 32-37 cells, error bars are 95% CI, $p = 0.0002$, two-tailed t-test.
