# Supplementary material for: Complete microtubule–kinetochore occupancy favours the segregation of merotelic attachments
Source: Nat Commun. 2018 May 23;9:2042. doi: 10.1038/s41467-018-04427-x (PMC5966435; doi:10.1038/s41467-018-04427-x)
Supplement: Supplementary file 3 — Description of Additional Supplementary Files [file 41467_2018_4427_MOESM3_ESM.docx]

**Description of Additional Supplementary Files**

File Name: Supplementary Movie 1

Description: Kinetochore oscillations in control-treated cell: Exemplary hTertRPE1

GFP-CENPA metaphase cell treated with DMSO and used for automated sister-KT tracking

analysis. Images were taken every 15 s. Scale bars = 5 µm.

File Name: Supplementary Movie 2

Description: Kinetochore oscillations in BAL27862-treated cell: hTert-RPE1

GFP-CENPA metaphase cell treated with 12 nM BAL27862 and used for automated sister-KT

tracking analysis. Images were taken every 15 s. Scale bars = 5 µm

File Name: Supplementary Movie 3

Description: Mitotic progression in control-treated cell: Exemplary hTert-RPE1

H2B-mCherry/EB3-eGFP cell treated with DMSO. Images were taken every 3 min. Scale bars = 10 µm

File Name: Supplementary Movie 4

Description: Mitotic progression in BAL27862-treated cell: Exemplary hTertRPE1

H2B-mCherry/EB3-eGFP cell treated with 12 nM BAL27862. Images were taken every 3

min. Scale bars = 10 µm.
